# Design and Experimental Validation of a Multimodal Snake Robot with Elliptical Wheels

**DOI:** 10.3390/biomimetics10080532

**Published:** 2025-08-13

**Authors:** Xuan Xiao, Zizhu Zhao, Lianzhi Qi, Michael Albert Sumantri, Hengwei Liu, Jianqin Li, Keyang Zheng, Jianming Wang

**Affiliations:** 1 School of Computer Science and Technology, Tiangong University, Tianjin 300387, China; xxiao@tiangong.edu.cn (X.X.); superdragonlcl7@gmail.com (Z.Z.); 2 School of Mechanical Engineering, Tiangong University, Tianjin 300387, China; qilianzhi774@163.com; 3 School of International Education, Tiangong University, Tianjin 300387, China; michaelalbert752@gmail.com; 4 School of Electronic and Information Engineering, Tiangong University, Tianjin 300387, China; liuhengwei@tiangong.edu.cn (H.L.); llhh085243@163.com (J.L.); zhengkeyang339@outlook.com (K.Z.); 5 Tianjin Key Laboratory of Autonomous Intelligence Technology and Systems, Tiangong University, Tianjin 300387, China

**Keywords:** snake robot, multimodal locomotion, body-based locomotion, wheeled locomotion, wheel-body coordinated locomotion

## Abstract

Snake robots are characterized by their flexibility and environmental adaptability, achieved through various optimized gaits. However, their forward propulsion still requires improvement. This challenge can be addressed by integrating wheels or legs, but these mechanisms often limit the ability of snake robots to perform most optimized gaits. In this article, we develop a novel multimodal snake robot, JiAo-II, with both body-based locomotion and wheeled locomotion to handle complex terrains. The mechanical design and implementation of JiAo-II are presented in detail, with particular emphasis on its innovative elliptical wheels and gear transmission mechanism. Experimental results validate the effectiveness and multifunctionality of JiAo-II across various scenarios, including traversing grasslands, crossing gaps, ascending slopes, navigating pipelines, and climbing cylindrical surfaces. Furthermore, a series of experiments are conducted to evaluate the performance of the wheel–body coordinated locomotion on uneven ground, demonstrating the robustness even without requiring external sensing or sophisticated control strategies. In summary, the proposed multimodal mechanism significantly enhances the locomotion speed, terrain adaptability and robustness of snake robots.

## 1. Introduction

Snake robots, due to their slender and flexible structures, exhibit excellent maneuverability and adaptability to diverse terrains, demonstrating significant potential for applications in complex environments such as search-and-rescue missions, pipeline inspection, minimally invasive surgery and space exploration. However, the movement speed and efficiency of current snake robots are considerably lower than those of biological snakes, primarily due to the absence of muscle-scale contraction mechanisms. To improve the overall performance of snake robots, researchers have primarily focused on two strategies: one involves optimizing gait patterns through control algorithms, while the other emphasizes hardware structural improvements by integrating wheeled, tracked or legged components.

In terms of gait optimization, researchers have primarily focused on designing new body-based locomotion patterns to enhance the flexibility and environmental adaptability of snake robots. For example, Lipkin et al. proposed differentiable and piecewise-differentiable gaits to improve motion control performance in snake robots [1]. Chirikjian and Burdick generated motion gaits based on a continuous backbone curve model [2]. The ACM-III and ACM-R5 robots, developed by Yamada and Hirose, simulated the movement patterns of natural snakes, effectively improving stability and forward speed on flat terrain [3,4]. Takemori et al. proposed various adaptive gait generation methods for snake robots in complex environments [5,6]. Ono et al. developed a snake-like versatile and intelligent robot designed to access the subsurface oceans of icy moons by descending into erupting vents [7]. In addition, researchers have also developed specialized gaits to achieve specific locomotion tasks, such as straight-line progression, climbing, and obstacle negotiation [8,9,10]. Although these methods have achieved positive results in enhancing robot mobility, generating precise and robust gait patterns in complex and dynamic environments remains a significant challenge.

On the other hand, hardware structural improvements primarily focuses on enhancing locomotion speed and stability. For instance, Hirose and Morishima proposed a practical mobile robot with an articulated body, specifically designed for nuclear reactor inspection [11]. Fjerdingen et al. developed a wheeled, snake-like robot (PIKo) designed for the internal inspection of complex pipe structures, where the wheels provide additional propulsion to enhance mobility in confined environments [12]. The ACM-R4 robot, developed by the team led by Takaoka and Kouno, incorporates active wheels on both sides of its body, significantly enhancing its obstacle-crossing capability and terrain adaptability [13,14]. In addition to wheeled locomotion, tracked and legged systems have been introduced, significantly improving the movement capabilities of snake robots in complex environments [15,16]. Despite these structural improvements, such modifications often prevent snake robots from performing most body-based locomotion.

To combine the advantages of gait optimization and hardware improvements, we proposed a wheeled snake robot named JiAo. The robot featured an innovative elliptical wheel mechanism while maintaining a cylindrical body structure, enabling smooth transitions between wheeled locomotion and body-based locomotion. JiAo demonstrates efficient and flexible locomotion capabilities in various scenarios. However, the robot frequently tips over on uneven ground, highlighting the need to improve its stability and robustness.

In addition to snake robots, centipede-inspired robots have also garnered significant attention and research interest in recent years. These robots enhance locomotion performance in complex environments through coordinated, rhythmic movements of multiple body segments and legs, while also providing insights into the locomotion mechanisms of biological centipedes and other arthropods. For example, Chong et al. proposed a general control framework for identifying locomotion templates in serially connected, multi-legged robots [17]. They extended their low-dimensional geometric analysis to illustrate how body undulation improves performance on uneven and obstacle-rich terrains. This framework was also applied to quantitatively model the locomotion dynamics of biological centipedes—specifically the desert centipede Scolopendra polymorpha [18]. Experimental results confirmed that sufficient spatial redundancy, as achieved in serially connected legged robots, enables reliable locomotion in complex environments, such as collapsed buildings or crop fields, without requiring external sensing or sophisticated control strategies [19]. In summary, by templating the movement mechanism of serially connected multi-legged robots, the motion performance of JiAo will be effectively improved.

**Figure 1:**
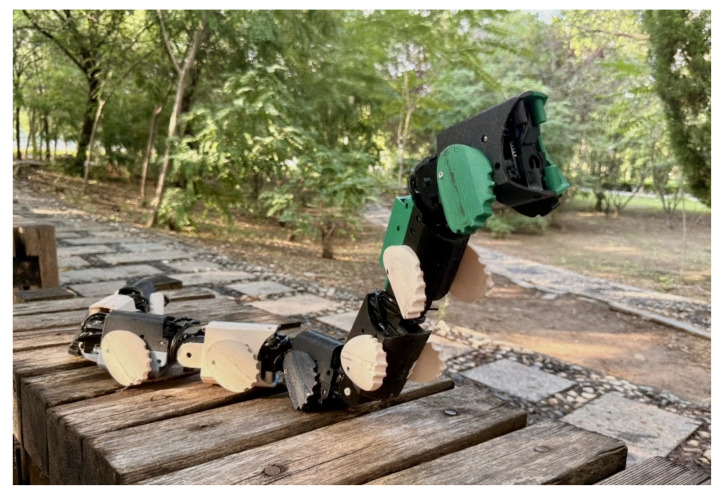
Snake robot with elliptical wheels.

To address the aforementioned issues, this article introduces the novel JiAo-II as shown in Figure 1, based on previous research. It improves the robot’s support stability and wheel–motor transmission mechanism, which enhances stability and obstacle-crossing capability. Additionally, the wheel–body coordinated locomotion is further explored to enhance the robot’s motion robustness. In summary, the main contributions of this article are as follows.

- Development of a Versatile Snake Robot: This study presents an improved design for a novel snake robot, JiAo-II, which integrates the advantages of both wheeled locomotion and body-based locomotion. The elliptical wheel mechanism retains the cylindrical profile of the robot body while enabling efficient self-propelled motion. Moreover, it allows seamless transitions between different locomotion modes, significantly enhancing the robot’s adaptability to diverse terrain conditions.
- Experimental Validation and Performance Evaluation: A comprehensive experimental platform was established to systematically evaluate the performance of JiAo-II across various typical tasks, including grass traversal, gap crossing, slope ascending, pipe navigation, and cylindrical climbing. In addition, the influence of key parameters, such as the wheel duty cycle and horizontal and vertical body undulations, on locomotion speed and stability was analyzed. Experimental results demonstrate that JiAo-II exhibits strong terrain adaptability and holds significant potential for practical engineering applications.

## 2. Mechanical Design and Implementation

### 2.1. Design Concept

By integrating the advantages of traditional snake robots in rapidly adapting to complex terrains and the strong locomotion stability of wheeled snake robots on smooth terrains, the design requirements for the new multimodal snake robot are as follows.

- The design of the snake wheels should align with the robot’s originally defined cylindrical body, without causing significant alterations to its overall external shape.
- During motion, the contact point of the wheels with the ground should be located below the robot’s body, allowing the wheels to lift the entire or part of the body off the ground.
- To meet the constraints of limited internal space, the robot’s transmission system must be compactly arranged while ensuring efficiency and reliability.
- To ensure dynamic stability during wheeled locomotion, the structural design should provide a sufficiently large support area between the robot and the ground, effectively preventing tipping or instability.

Traditional circular wheels clearly struggle to meet all of these requirements simultaneously. Inspired by the design principles of RHex [20], we propose a novel structural design in which the robot incorporates a shell with an inclined surface and elliptical wheels featuring grooves. When the wheels on both sides are in a specific phase, the robot’s cross-section, when viewed from the front, retains the original circular profile. Upon wheel rotation, the inclined surface design creates a fixed angle between the two wheels, achieving the required stability while also meeting the above design objectives. This design effectively combines the stability and efficiency of wheeled locomotion with the flexibility and environmental adaptability of body-based locomotion, achieving a synergistic integration of both modes. As shown in Table 1, a comparison with other snake robots demonstrates that the proposed robot exhibits significant advantages in executing diverse locomotion gaits. Specifically, the wheeled configuration substantially improves the robot’s speed and stability when traversing gaps, offering notable performance improvements over the wheelless design proposed by Takemori et al. Moreover, some existing robots are structurally constrained by the integration of wheels, legs, or tracks, limiting their ability to perform complex motions such as helical rolling inside pipes or climbing cylindrical surfaces. In contrast, the proposed robot successfully accomplishes all these tasks without compromising flexibility. In summary, this design meets the core performance requirements of advanced hybrid robots and lays a solid foundation for future improvements in motion control and autonomous navigation over complex terrains.

### 2.2. Module Design and Implementation

**Figure 2:**
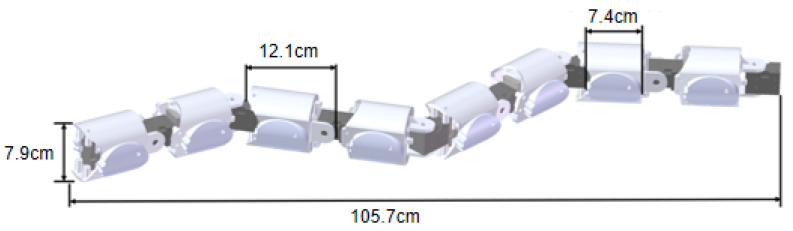
Configuration diagram of the elliptical wheel snake robot.

**Figure 3:**
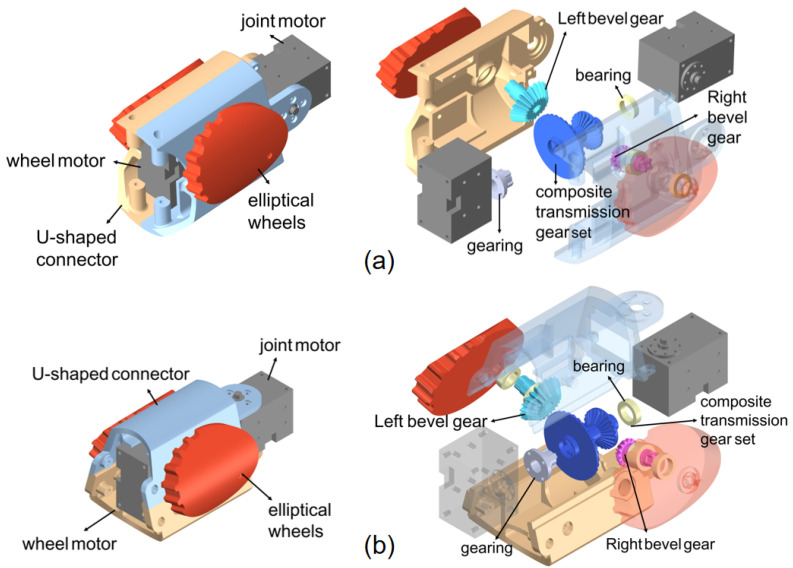
Different unit modules. (**a**) Pitch module. (**b**) Yaw module.

The snake robot proposed in this article adopts an advanced modular serial design concept, as shown in Figure 2. Each modular unit consists of a U-shaped connector, a joint motor, a wheel motor, and two symmetrically arranged elliptical wheels. The internal transmission system is primarily composed of bevel gears and several bearings. These components work synergistically, and the modules are connected via joint motors, ensuring that adjacent modules can rotate flexibly. Additionally, the two adjacent unit modules consist of a pitch module and a yaw module, respectively, and these two modules are orthogonally arranged in the robot’s configuration, thereby enabling the simulation of the unique locomotion patterns observed in natural snakes. The detailed design of these two modules is shown in Figure 3. The joint motors and wheel motors used are both Dynamixel XH430-W350-R models, and they operate in position control mode. Meanwhile, the U-shaped connector, bevel gears, composite transmission gears, bearings, and elliptical wheels are all 3D printed, with the gears fabricated from resin material to ensure high mechanical strength and durability, further enhancing the robustness and reliability of the components.

**Figure 4:**
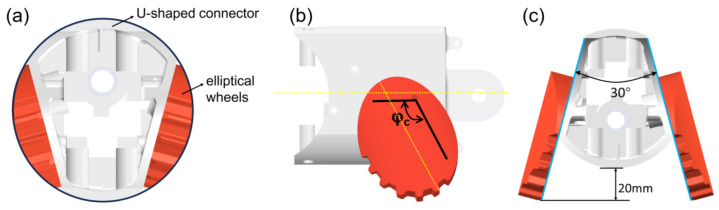
Design of the robot’s body shell and wheels. (**a**) When the wheel motors are in the initial phase, the robot’s cross-sectional profile. (**b**) $\varphi_{c}$ denotes the angle traversed by the wheel motors of the elliptical wheels. (**c**) The inclined shell design establishes a fixed angular offset between the two elliptical wheels. This configuration improves the robot’s rollover resistance.

To satisfy the design requirements for both external shape and ground contact performance, an inclined robotic body shell integrated with elliptical wheels is proposed. When the major axes of the elliptical wheels on both sides are aligned parallel to the body, i.e., when the wheel motors are in the initial phase, the robot’s cross-sectional profile remains circular, as shown in Figure 4a. This allows the robot to maintain its original cylindrical shape during the rolling motion. This feature ensures the robot maintains its inherent terrain adaptability. Additionally, the angle traversed by the wheel motors of the elliptical wheels is denoted as $\varphi_{c}$, as shown in Figure 4b. Furthermore, the lowest point of the elliptical wheels is significantly below the bottom of the robot’s main body. When the elliptical wheels complete one full rotation, their raised portions can lift the robot forward, creating a ’stepping’ effect that substantially enhances its obstacle-clearing ability. Moreover, during locomotion, the inclined shell design establishes a fixed angular offset between the two elliptical wheels. This configuration improves the robot’s rollover resistance and significantly enhances its overall stability while moving, as illustrated in Figure 4c.

**Figure 5:**
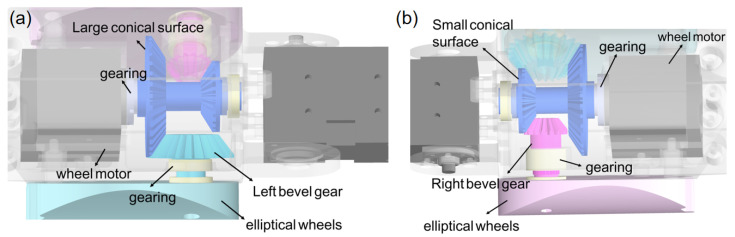
Assembly rendering of the transmission system. (**a**) Transmission system of the left elliptical wheel in a single module. (**b**) Transmission system of the right elliptical wheel in a single module.

Due to the limited internal space within the robotic body, conventional spur gears are unable to achieve the desired final transmission effect. Therefore, bevel gears are employed to enable both the redirection of the rotational axis and the synchronized rotation of the two wheels. The power transmission process involves the composite transmission gear meshing with two separate bevel gears via its two conical surfaces with different parameters, thereby driving the two elliptical wheels in synchronization. The specific transmission mechanism involves the connection of the output shaft of the wheel motor to the main shaft of the composite gear via a transmission mechanism. The large-end conical surface of the composite gear engages with the right-side bevel gear, while the small-end conical surface engages with the left-side bevel gear. In particular, although the gear ratios of both pairs of bevel gears are designed as 3:2, differences in the module and number of teeth on the two conical surfaces of the composite gear allow for precise mesh contact that ensures consistent rotational speed of the two wheels. This design enables synchronized control of the elliptical wheels while meeting the specific motion coordination requirements of the system. The detailed spatial configuration is illustrated in Figure 5.

Although this wheeled structure somewhat reduces the movement efficiency typically associated with traditional wheeled robots, it retains the core design features of the snake-like robot, not only maintaining its superior obstacle-surpassing ability and stability in complex terrains but also improving its movement speed.

### 2.3. System Design and Implementation

Based on the mechanical structure design, this study further develops the communication and power supply systems to realize the overall functionality of the robot. The following outlines the specific implementation of each system.

**Figure 6:**
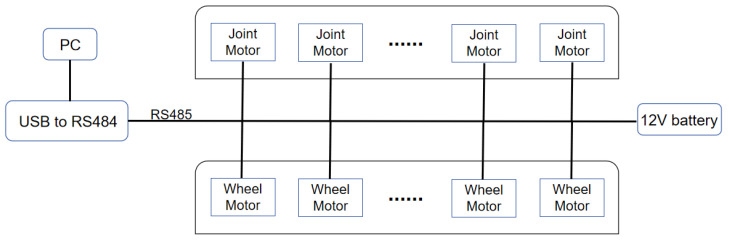
Power and communication system.

As previously mentioned, both the joint motors and wheel motors use the same model of drive modules; thus, the RS-485 communication protocol is adopted for control. The system architecture is illustrated in Figure 6. To facilitate data exchange between the host computer (PC) and the robot, a USB-to-RS485 conversion module is integrated, ensuring efficient transmission and execution of commands. The entire system is powered by a single 12V lithium battery, which provides stable energy support for all joint and wheel motors. This configuration simplifies the power management structure and enhances the system’s integration.

**Figure 7:**
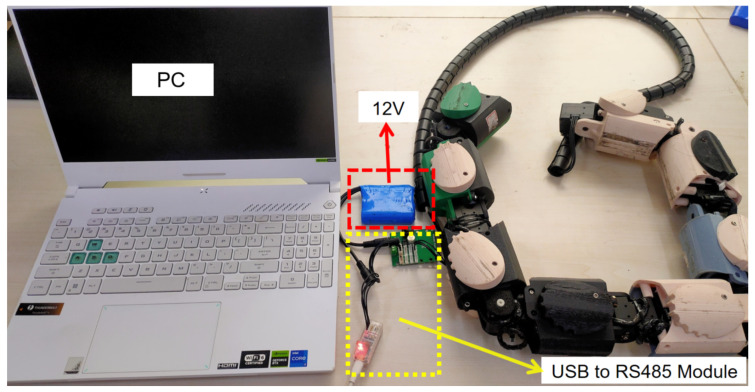
The experimental platform.

To comprehensively evaluate the performance of JiAo-II, an experimental platform was constructed. This platform includes the controller, power supply system, and the snake robot itself, as shown in Figure 7. The control system is centered on a host computer (a personal computer, PC), specifically an ASUS laptop equipped with an Intel i7-13620H processor, 16 GB of RAM, a 512 GB solid-state drive, and an RTX 4060 GPU. The PC runs the control algorithm and transmits motion commands to the robot. A signal converter is employed to transform USB signals from the PC into control signals compatible with the communication protocol, which are then transmitted to the Dynamixel servo motors. Table 2 summarizes the main parameters of the robot for subsequent analysis.

## 3. Control Law

### 3.1. Wheeled Locomotion Control

Inspired by Hildebrand’s symmetric quadruped gait theory [21], this study extends the theory to the wheeled snake robot system and models and analyzes the motion patterns of JiAo-II. The core of this model relies on two key parameters: the duty factor (D) and the lateral phase lag ($\Phi_{\mathrm{lat}}$).

The duty factor (D) represents the fraction of the gait period during which each leg is in contact with the ground, with values ranging from 0 to 1. The lateral phase lag ($\Phi_{\mathrm{lat}}$) represents the degree of lag between adjacent forelimb and hindlimb movements. When *T* represents the total duration of the gait period, and $T_{d}$ represents the time by which the ground contact of the hindlimb lags behind that of the forelimb, $\Phi_{\mathrm{lat}}$ can be calculated using the following formula.

(1) $$\Phi_{\mathrm{lat}}=\frac{T_{d}}{T}$$

Key Assumptions: Based on the core assumptions of Hildebrand’s symmetric gait model, and considering the structural characteristics of the robot system in this study, the following applicable conditions are proposed:

- All wheels have the same duty factor.
- The motion phases of the corresponding legs on the left and right sides are synchronized.
- The lateral phase lag is consistent between the left and right sides.

These assumptions not only simplify the control logic but also provide a theoretical foundation for the synchronization and coordination of the multi-wheel system.

Contact State Modeling: To describe the robot’s contact states under different gait phases, we introduce a binary variable *c* to represent the state of each wheel:

(2) c=1:StancePhase(supporting)c=0:SwingPhase(non‐supporting)

For a specific gait phase $\varphi_{c}$, the contact state $c_{l}\left(\varphi_{c},i\right)$ of the *i*-th wheel on the left side can be expressed as follows:

(3) $$c_{l}\left(\varphi_{c},1\right)=\left\{\begin{array}{cc} 1, & \mathrm{if}\;\;\mathrm{mod}\;(\varphi_{c},2\pi )<2\pi D\\ 0, & \mathrm{otherwise} \end{array}\right.$$

For subsequent wheels, their contact state is recursively defined as follows:

(4) $$c_{l}\left(\varphi_{c},i\right)=c_{l}\left(\varphi_{c}+2\pi \left(i-1\right)\Phi_{\mathrm{lat}},1\right)$$

$c_{l}\left(\varphi_{c},i\right)$ and $c_{r}\left(\varphi_{c},i\right)$ represent the contact state of the *i*-th wheel on the left and right side at the gait phase $\varphi_{c}$, respectively.

(5) $$c_{r}\left(\varphi_{c},i\right)=c_{l}\left(\varphi_{c},i\right)$$

**Figure 8:**
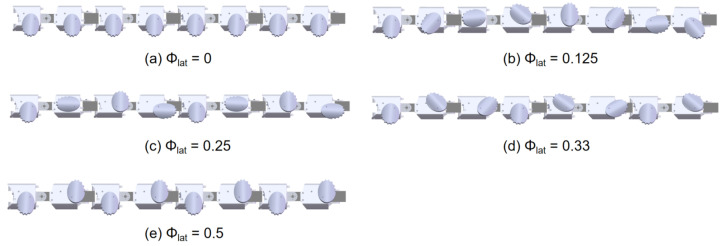
Different wheeled locomotion mode.

Through this model, various motion patterns of the robot in complex terrains can be precisely modeled and controlled. In the experiments of this study, several configurations of lateral phase lag were considered, as shown in Figure 8, where the phase of the first wheel is $\varphi_{c}=90^{\circ }$. These configurations include the 1-group mode with $\Phi_{\mathrm{lat}}$ = 0; the 2-group mode, where the wheels are divided into odd and even groups with $\Phi_{\mathrm{lat}}$ = 0.5, forming an alternating support gait similar to RHex; the 3-group mode, where the wheels are divided into three groups with $\Phi_{\mathrm{lat}}$ = 0.33, enhancing stability in support; the 4-group mode, where the wheels are divided into four groups with $\Phi_{\mathrm{lat}}$ = 0.25, suitable for medium to high-speed traversal in complex terrain; and the 8-group mode, where the wheels are divided into eight groups with $\Phi_{\mathrm{lat}}$ = 0.125, achieving more refined support distribution, ideal for high-precision control or extreme terrain environments.

### 3.2. Body-Based Locomotion Control

Due to its cylindrical body design, JiAo-II retains most of the locomotion abilities typical of body-based locomotion. Examples of these capabilities include the Helix Rolling Gait, Lateral Rolling Gait, and Compliant Control.

#### 3.2.1. Helix Rolling Gait

Helix Rolling Gait is a common locomotion method for robots with cylindrical or similar body structures. The robot moves by rolling along a helical trajectory, inspired by the way some animals or mechanical systems, such as worms or certain bio-inspired robots, use helical motions for forward movement. In this gait, the robot’s movement follows a spiral path, typically composed of both lateral and rotational motions. The robot rolls its body on the ground, generating forward or lateral displacement, and its overall trajectory forms a three-dimensional helical curve rather than a simple straight line or arc. This method generally relies on the geometric properties of the backbone curve and determines the robot’s configuration through classical kinematic principles.

A key mathematical tool in this control strategy is the Frenet–Serret framework, which defines three orthogonal basis vectors:

- $e_{1}\left(s\right)$: Tangent vector, representing the direction of the curve’s tangent.
- $e_{2}\left(s\right)$: Normal vector, representing the direction of the curve’s normal.
- $e_{3}\left(s\right)$: Binormal vector, which is the cross product of $e_{1}\left(s\right)$ and $e_{2}\left(s\right)$.

In the robot’s coordinate system, $e_{r}\left(s\right)=e_{1}\left(s\right)$, and $e_{p}\left(s\right)$ and $e_{y}\left(s\right)$ correspond to the robot’s pitch and yaw axes, respectively. The twist angle ψ(s), which represents the rotation between the Frenet–Serret frame and the robot’s backbone frame, is defined as follows:

(6) $$\psi \left(s\right)=\int_{0}^{s}\tau \left(\hat{s}\right)\;d\hat{s}+\psi_{0}$$

where $\tau (\hat{s})$ is the torsion at arc length *s*, indicating the rate of change in the curve’s orientation, and $\psi_{0}$ is an arbitrary constant that adjusts the initial twist angle, allowing the robot’s backbone to rotate, thus generating rolling motion.

The desired joint angles $\theta_{i}^{d}$ for the robot are calculated based on the curvature κ(s) and the twist angle ψ(s) as follows:

(7) $$\theta_{i}^{d}=\left\{\begin{array}{cc} \int_{s_{h}-\left(i+1\right)l}^{s_{h}-\left(i-1\right)l}-\kappa \left(s\right)\sin\psi \left(s\right)\;ds & \mathrm{for}\;\mathrm{odd}\;\;i,\\ \int_{s_{h}-\left(i+1\right)l}^{s_{h}-\left(i-1\right)l}\kappa \left(s\right)\cos\psi \left(s\right)\;ds & \mathrm{for}\;\mathrm{even}\;\;i. \end{array}\right.$$

Here, $s_{h}$ represents the position of the robot’s head along the target curve. By adjusting $s_{h}$, shift control is achieved, enabling the robot’s body to follow the target curve precisely. By controlling the curvature and twist angle along the curve, the snake robot can achieve coordinated, body-based locomotion that matches its desired target shape.

This method not only allows the snake robot to follow complex curved paths accurately but also enables it to perform locomotion without wheels, showcasing its adaptability in various terrains.

#### 3.2.2. Lateral Rolling Gait

The Lateral Rolling Gait [22] is typically achieved through the coordinated movement of the robot’s joints. It combines rotational and rolling motions to enable effective movement on a plane. The key to this gait is the rotation of certain joints, which generates relative displacement while maintaining system stability. The lateral rolling gait can be succinctly represented by the following formula:

(8) $$\alpha \left(t,i\right)=\left\{\begin{array}{cc} \frac{2l}{R}\cos\left(\omega t\right) & \mathrm{for}\;\mathrm{odd}\;i,\\ \frac{2l}{R}\sin\left(\omega t\right) & \mathrm{for}\;\mathrm{even}\;i. \end{array}\right.$$

In this formulation, α(t,i) represents the angular displacement of the robot’s body segment at time *t* and index *i*, where *l* denotes the length of the segment, and *R* is the radius that determines the curvature of the rolling motion. The parameter ω controls the oscillation frequency, thereby adjusting the speed of the robot’s lateral rolling. The alternating sine and cosine terms for even and odd segments ensure that the robot’s motion is coordinated, with distinct rolling phases for each body part. This approach allows for smooth lateral movement, with the curvature *R* dictating the shape of the motion, enabling the robot to maneuver efficiently across different surfaces.

#### 3.2.3. Compliant Control

Compliant control [23,24] is a widely used technique in robotic control, aimed at enabling robots to interact gently with objects or surfaces in their environment. This control strategy is particularly useful for tasks that require interaction with uncertain or dynamic environments, such as object grasping, assembly, and surgical robotics. Unlike traditional rigid control methods, which maintain a fixed posture and force during robot motion, compliant control allows the robot to exhibit some flexibility when in contact with external objects. This flexibility enables the robot to adapt to external force variations, reducing the risk of impact or damage to both the robot and the object being interacted with.

Compliant control typically relies on force–position or force–velocity feedback loops to regulate the robot’s motion. The key aspect of this control method is that the robot adjusts its motion based on the forces exerted by the objects it contacts, thereby achieving a soft and adaptive interaction with the environment.

Compliant control can be implemented using the robot’s dynamic model. Let the robot’s mass matrix be denoted by M(θ), and the control torque by τ. The dynamics of compliant control can be represented by the following equation:

(9) $$M\left(\theta \right)\ddot{\theta }+C\left(\theta ,\dot{\theta }\right)\dot{\theta }+G\left(\theta \right)=\tau$$

where:

- θ is the joint angle of the robot,
- M(θ) is the mass matrix of the robot,
- $C(\theta ,\dot{\theta })$ is the Coriolis force term,
- G(θ) represents the gravitational term,
- τ is the control torque applied to the robot.

By adjusting the control torque τ, compliant control allows the robot to adapt to external forces and dynamically adjust its motion.

In summary, compliant control enhances a robot’s ability to respond to changes in the environment, enabling it to perform tasks with greater flexibility and adaptability. This makes it an essential tool in applications requiring interaction with uncertain or dynamic environments, such as collaborative robotics, medical surgery, and automated assembly.

### 3.3. Wheel–Body Coordinated Locomotion Control

**Figure 9:**
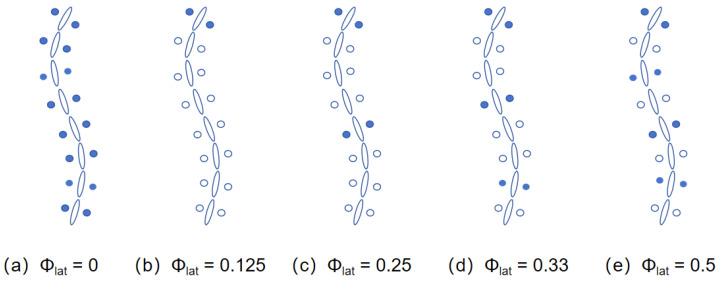
Different wheel–body coordinated locomotion mode.

Wheel–body coordinated locomotion control introduces horizontal and vertical body undulations into wheeled locomotion across different $\Phi_{\mathrm{lat}}$ configurations, as illustrated in Figure 9. Specifically, the formula for body undulation can be expressed as a time-dependent equation:

(10) $$\varphi_{i}\left(t\right)=\left\{\begin{array}{cc} A_{e}\sin\left(w_{e}t+iv_{e}\right)+\lambda_{e} & \mathrm{for}\;\mathrm{even}\;i,\\ A_{o}\left|\sin\left(w_{o}t+iv_{o}\right)\right|+\lambda_{o} & \mathrm{for}\;\mathrm{odd}\;i. \end{array}\right.$$

where the subscripts *e* and *o* denote parameters for even and odd modules, respectively:

- $A_{e},A_{o}$ Amplitude for even and odd modules, respectively;
- $w_{e},w_{o}$ Angular frequency for even and odd modules, respectively;
- $v_{e},v_{o}$ Phase modulation coefficient for even and odd modules, respectively;
- $\lambda_{e},\lambda_{o}$ Offset or baseline shift for even and odd modules, respectively.

By adjusting these parameters, the body undulation gait can be generated.

## 4. Experiments

To verify and test the robot’s characteristics, we conducted a series of experiments, including the wheeled locomotion experiment, body-based locomotion experiment, and wheel–body coordinated locomotion experiment.

### 4.1. Wheeled Locomotion Experiment

#### 4.1.1. Wheeled Locomotion on Different Terrains

**Figure 10:**
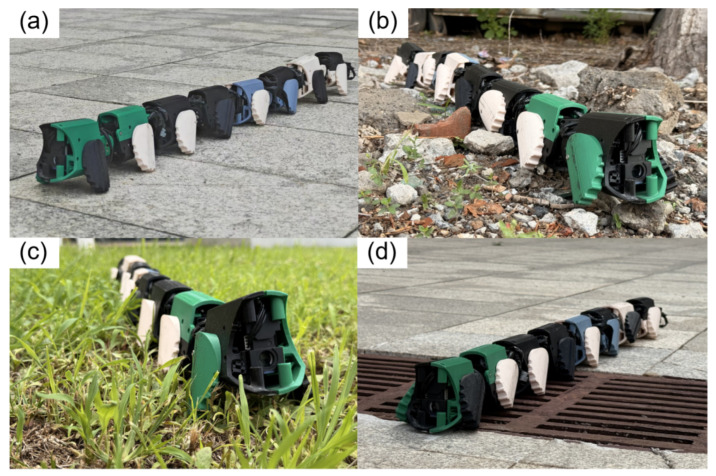
(**a**) The robot traversed the cement road. (**b**) The robot navigated along a rocky gravel path. (**c**) The robot walked on the grass. (**d**) The robot traversed the drainage grates.

This experiment systematically evaluated the movement performance of the JiAo-II robot across four typical outdoor terrains, which included cement pavement, gravel road, grass, and drainage grids, as shown in Figure 10. To comprehensively assess the robot’s adaptability in complex environments, we tested its movement speed under various locomotion modes. To ensure the accuracy and repeatability of the experimental data, clear start and end markers were established for each trial. The robot’s movement time was recorded from which the average travel speed was calculated.

**Figure 11:**
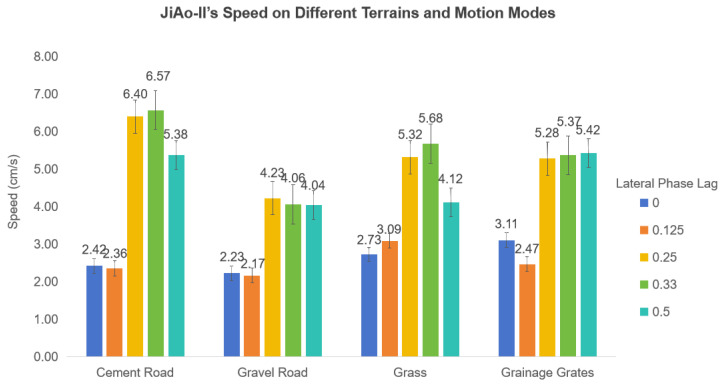
The moving speed of JiAo-II across four terrains (cement road, gravel road, grass, and drainage grates) was measured across different motion modes.

Speed Analysis: In consideration of potential random errors during the experiments, five repetitions were conducted for each terrain type and its corresponding locomotion mode. The arithmetic mean of the data from these repetitions was used as the final result, ensuring enhanced statistical reliability. The summarized results were presented in Figure 11, providing a clear representation of the variation in JiAo-II’s movement efficiency across different terrains and gait strategies.

Experimental results indicated that the robot exhibited relatively slower speeds under lateral phase lags of 0 and 0.125, primarily due to the lower contact frequency between the wheels and the ground, which led to reduced stability. In contrast, the robot achieved its highest speed at lateral phase lags of 0.25 and 0.33, where both the wheel duty cycle and system stability were maximized. At a lateral phase lag of 0.5, the speed decreased despite a high duty cycle, as the contact frequency between the wheels and the ground was significantly reduced. Moreover, the overall speed of the robot was lower on gravel roads and drainage grates compared to cement pavement and grass. This was mainly attributed to the increased slippage experienced by the wheels on these terrains, which compromised locomotion efficiency.

Energy Consumption Analysis: The energy consumption of robotic systems is influenced by various factors, including terrain conditions, locomotion speed and gait stability. When $\Phi_{\mathrm{lat}}$ is 0.125, 0.25, or 0.33, JiAo-II is prone to wheel slippage, leading to energy waste and reduced locomotion efficiency. Therefore, considering both motion stability and energy efficiency, we selected the gait with $\Phi_{\mathrm{lat}}=0.5$ for energy consumption analysis.

**Figure 12:**
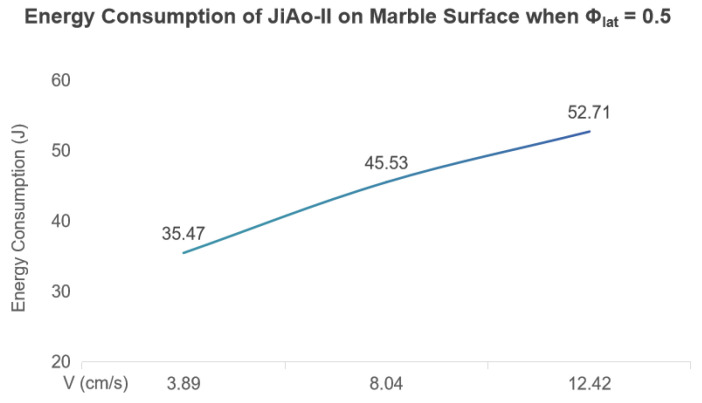
Energy consumption comparison of JiAo-II on marble surface across three locomotion speeds with $\Phi_{\mathrm{lat}}=0.5$.

Under this gait, we tested the robot’s energy consumption at three different average speeds 3.89 cm/s, 8.04 cm/s, and 12.42 cm/s. In each test, the robot operated continuously for 10 s and the total energy consumed during this period was recorded. The measured energy expenditures were 35.47 J, 45.53 J and 52.71 J, respectively, as shown in Figure 12.

To further evaluate locomotion efficiency, we adopted the Specific Resistance (SR) as the evaluation metric [25], defined as follows:

(11) $$\mathrm{SR}=\frac{P}{mgv}$$

where *P* is the average power (in W = J/s), mg is the robot’s total weight (in N), and *v* is the average forward speed (in m/s).

Analysis based on the SR model shows that although the total energy consumption increases with speed over the same duration, the SR value significantly decreases due to the improved motion efficiency at higher speeds, indicating that the energy utilization efficiency is actually enhanced. This demonstrates that JiAo-II achieves better energy efficiency at higher speed ranges. Furthermore, at a speed of 12.42 cm/s and powered by a 1000 mAh lithium battery, JiAo-II can maintain continuous movement on the treadmill for at least one hour with $\Phi_{\mathrm{lat}}=0.5$.

#### 4.1.2. Gap Crossing

**Figure 13:**
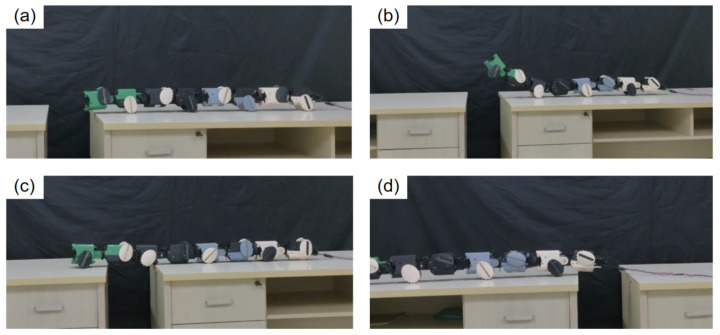
(**a**) The robot approached the gap using wheeled locomotion. (**b**) The robot lifted its head. (**c**) After crossing the gap, the robot lowered its head joint. (**d**) The robot successfully completed its passage over the gap.

Leveraging the forward motion provided by its wheeled drive motors, JiAo-II demonstrated exceptional obstacle-crossing capability. To assess this performance, a gap-crossing experiment was designed and conducted, with the results shown in Figure 13. The experiment aimed to simulate typical outdoor obstacles, such as cliffs or ditches, to evaluate JiAo-II’s ability to adapt to sudden terrain changes.

In this test, two platforms of equal height were separated to form a 20 cm gap, representing a localized loss of support commonly encountered in real-world environments. JiAo-II successfully crossed the gap using the 2-group wheeled locomotion mode. The crossing process was divided into the following stages.

- Initial Phase (Figure 13a): JiAo-II began moving steadily towards the gap in wheeled locomotion mode.
- Approaching the Gap (Figure 13b): As the robot neared the edge, the front joints actively raised in preparation for the crossing. The unlifted elliptical wheels continued to provide propulsion, moving the robot forward and shifting its center of gravity for the transition.
- Transition Phase (Figure 13c): Once the first module successfully crossed the gap and landed, it quickly returned to its normal support state, followed by the remaining modules crossing in sequence.
- Completion Phase (Figure 13d): The robot resumed its wheeled locomotion mode, completing the task.

This experiment validated JiAo-II’s ability to stably traverse disrupted terrains, particularly those with discontinuous support surfaces. By using pre-programmed control to coordinate its joint movements with the wheeled drive system, JiAo-II maintained continuous and stable motion, demonstrating strong adaptability in complex environments.

### 4.2. Body-Based Locomotion Experiment

#### 4.2.1. Cylindrical Climbing

**Figure 14:**
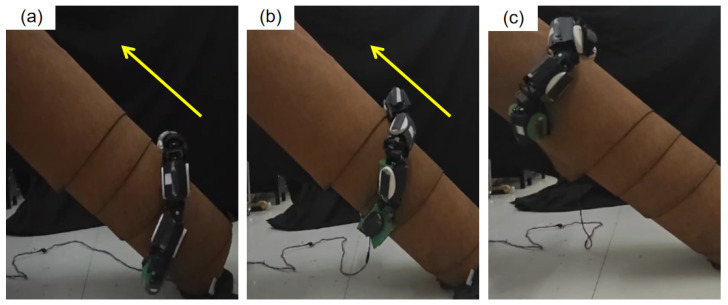
(**a**) The robot coiled around the cylinder, beginning its ascent. (**b**) The robot moves upward. (**c**) The robot reached the top at a speed of 3.05 cm/s. The arrows indicate the robot’s direction of motion.

To thoroughly evaluate JiAo-II’s ability to inherit the lateral rolling gait, we designed and implemented climbing experiments. As shown in Figure 14, the experiments were conducted using a plastic cylinder with a diameter of 20 cm, whose surface was covered with carpet to increase the coefficient of friction and more closely mimic the contact characteristics of real-world environments. The focus of these tests was to assess JiAo-II’s climbing performance both on a vertical surface and at a 41° inclined angle. To ensure the safety of the experiment and prevent mechanical damage due to instability or loss of control, we incorporated a compliant control strategy during the experiment to dynamically adjust joint torques and provide overload protection.

When JiAo-II attempted to climb the vertical cylinder. However, the robot failed to reach the top due to insufficient torque from the joint motors, causing it to slide down. In future work, we will attempt to increase the output torque of the joints and reduce the weight to address this issue. Subsequently, we adjusted the experimental setup by tilting the cylinder to a 41° angle, thus reducing the direct impact of gravity on the climbing process. Under these conditions, JiAo-II only needed to overcome a portion of the gravitational component and the frictional force from the contact surface. By employing a lateral rolling gait strategy, the robot successfully completed the climbing task in 40 s, achieving an average speed of 3.05 cm/s.

In conclusion, although JiAo-II was unable to achieve satisfactory results in the vertical climbing scenario due to the limitations of the current motor system, the experiment on the inclined cylinder successfully validated the effective inheritance and application of the lateral rolling gait. This demonstrated JiAo-II’s potential for stable movement in unstructured environments.

#### 4.2.2. Pipe Navigation

In order to gain an in-depth understanding of JiAo-II’s multimodal locomotion capabilities in pipe environments, a series of pipeline navigation experiments were designed and implemented. These experiments aimed to assess the robot’s performance in traversing narrow pipes under different movement modes, particularly focusing on the helical rolling gait based on body deformation and the traditional wheeled drive mode. The experiments were designed to compare the speed differences between the two modes. To ensure the scientific validity of the results, all tests were conducted in a controlled and safe environment, with the robot’s speed maintained at a stable velocity.

**Figure 15:**
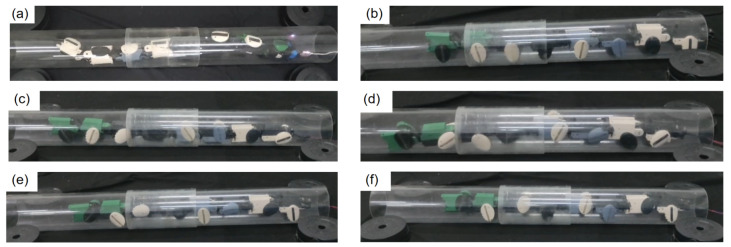
(**a**) The snake robot traversed the pipeline using body-based locomotion. (**b**) The snake robot traversed the pipeline using wheeled locomotion with $\Phi_{\mathrm{lat}}$ = 0. (**c**) The snake robot traversed the pipeline using wheeled locomotion with $\Phi_{\mathrm{lat}}$ = 0.125. (**d**) The snake robot traversed the pipeline using wheeled locomotion with $\Phi_{\mathrm{lat}}$ = 0.25. (**e**) The snake robot traversed the pipeline using wheeled locomotion with $\Phi_{\mathrm{lat}}$ = 0.33. (**f**) The snake robot traversed the pipeline using wheeled locomotion with $\Phi_{\mathrm{lat}}$ = 0.5.

The experiments were conducted on a standardized testing platform. A pipe with a length of 139 cm and an inner diameter of 15 cm was chosen as the experimental subject, simulating typical industrial pipe conditions. This setup was intended to thoroughly examine JiAo-II’s maneuverability and performance in confined spaces. Figure 15 illustrates the experimental setup.

In the first part of the experiment, JiAo-II’s performance using the helical rolling gait was tested. In this mode, JiAo-II adjusted its body shape to move forward, mimicking the flexible movement of snakes in narrow spaces. Specifically, JiAo-II utilized coordinated actions of its multiple joints to form a spiral shape, thereby propelling itself along the inner wall of the pipe. This method allowed the robot to adapt effectively to complex spatial constraints within the pipe. During the experiment, JiAo-II’s average forward speed in the helical rolling gait was 0.90 cm/s. Although this speed was relatively slow, the advantage lay in the ability to navigate complex environments without relying on wheeled propulsion, leveraging its body’s deformation to respond to environmental changes.

Subsequently, the robot was switched to the wheeled drive mode to compare its performance in this mode. In the wheeled drive mode, JiAo-II relied on integrated driving wheels to propel itself, aiming to increase its mobility efficiency and stability within the pipe. The experimental results were shown in Table 3, and the motion trend was consistent with that observed on flat ground. From the experimental results, it was evident that the wheeled drive mode demonstrated a significant speed advantage in traversing longer pipe distances and exhibited better stability and efficiency at higher speeds. However, the helical rolling gait offered greater flexibility, especially suitable for narrow or irregularly shaped pipe environments. Therefore, both modes had their respective advantages and could be selected based on the specific task requirements.

### 4.3. Wheel–Body Coordinated Locomotion Experiment

#### 4.3.1. Slope Climbing

**Figure 16:**
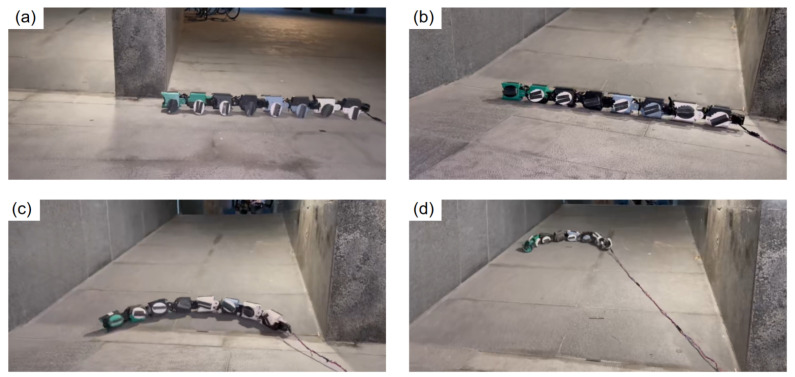
(**a**) The robot moved quickly using the wheel mode. (**b**) Upon reaching the target, the robot stopped and retracted its wheels. (**c**) The robot switched to rolling mode to ascend the slope. (**d**) The robot successfully climbed the slope.

To comprehensively evaluate the mobility and adaptability of JiAo-II across various terrain conditions, we designed and conducted a multimodal slope climbing experiment. During the experiment, JiAo-II initially employed a wheeled drive mode to efficiently traverse flat terrain. As it approached the slope, the system automatically switched to a rolling gait through pre-programmed control, enabling it to more effectively address the challenges posed by the inclined surface. The results of the experiment were shown in Figure 16.

The entire experiment was divided into two main phases. In the first phase, JiAo-II used its integrated wheel drive system to move at high speed across the flat terrain. The powerful thrust enabled JiAo-II to rapidly approach and prepare to climb a slope with a 30° inclination. This phase not only highlighted the efficiency of the wheeled drive mode on flat surfaces but also demonstrated its superior performance on structured terrain, confirming the advantages of wheeled drive in such environments. In the second phase, after JiAo-II reached the bottom of the slope, the system automatically switched to the rolling gait. By simulating the rolling behavior found in nature, JiAo-II successfully overcame the challenges posed by the slope. Ultimately, JiAo-II ascended to the peak of the slope at a speed of 2.28 cm/s, showcasing its exceptional adaptability and stability in unstructured terrains.

The results of the experiment demonstrated that JiAo-II was capable of flexibly switching between different motion modes based on terrain changes, effectively addressing the challenges posed by complex environments. This seamless transition between the wheeled drive and rolling gait not only enhanced JiAo-II’s passability in complex terrains but also validated the effectiveness of its multimodal motion control strategy, highlighting its outstanding terrain adaptability and flexibility.

Further analysis shows that when JiAo-II climbed a slope using the wheeled locomotion mode, it maintained a relatively high travel speed. However, the torque on the front joints increased significantly, often approaching or reaching the motor’s output limit, which introduces an overload risk. In contrast, the rolling gait maintained a moderate speed and resulted in a more uniform distribution of joint motor loads. The movement remained stable throughout the process, helping to reduce the load on individual motors, protect the drive units, and contribute to the overall safety and reliability of the robot’s operation. This indicates that the rolling gait represents a stable climbing strategy.

#### 4.3.2. Simple Obstacle Crossing

**Figure 17:**
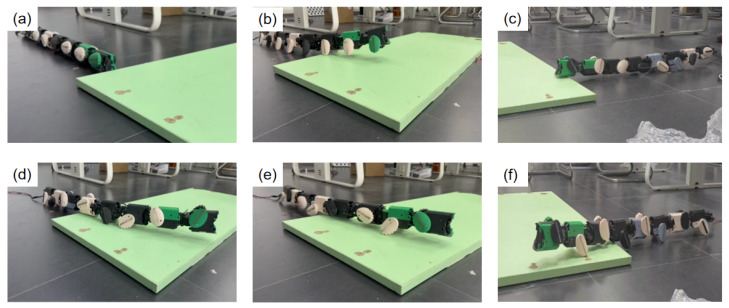
(**a**) A rectangular wooden board placed on a flat surface to simulate a sudden terrain obstacle. (**b**) The robot using the wheel-body coordinated locomotion with $\Phi_{\mathrm{lat}}$ = 0. (**c**) The robot using the wheel-body coordinated locomotion with $\Phi_{\mathrm{lat}}$ = 0.125. (**d**) The robot using the wheel-body coordinated locomotion with $\Phi_{\mathrm{lat}}$ = 0.25. (**e**) The robot using the wheel-body coordinated locomotion with $\Phi_{\mathrm{lat}}$ = 0.33. (**f**) The robot using the wheel-body coordinated locomotion with $\Phi_{\mathrm{lat}}$ = 0.5.

To evaluate the body adaptability of the JiAo-II snake robot when facing obstacles of fixed height, a set of obstacle-crossing experiments was designed. In the experiments, a rectangular wooden board with a thickness of 2.5 cm was placed on a flat surface to simulate a sudden terrain obstacle, as shown in Figure 17a. Preliminary tests indicated that without introducing body fluctuation strategies, the robot was unable to cross the obstacle. The reason for this was that in the pure wheeled locomotion mode, the highest point of the robot body supported by elliptical wheels was lower than the height of the obstacle, causing the front of the robot to fail in achieving sufficient vertical lift after contact with the obstacle, resulting in blockage and preventing further progress.

Based on this, body fluctuation strategies were further introduced, and systematic experiments were conducted for different wheel phase difference combinations, as shown in Figure 17b–f. Each phase difference condition was repeated five times to ensure statistical reliability of the data. The experimental results are shown in Table 4. In each experiment, the time taken by the robot to successfully cross the obstacle was recorded, and based on this, the average passing speed under each condition was calculated. The passing speed was derived from the time and distance traveled by the robot from the leading edge of the obstacle contact to the trailing edge after completely crossing the obstacle. The final speed result was averaged over the five experiments for performance comparison and analysis.

This experiment was conducted to verify the effectiveness of the body fluctuation strategy in overcoming local elevated terrain obstacles and to explore the impact of different wheel phase differences on the obstacle-crossing performance.

#### 4.3.3. Rough Terrain Traversal

**Figure 18:**
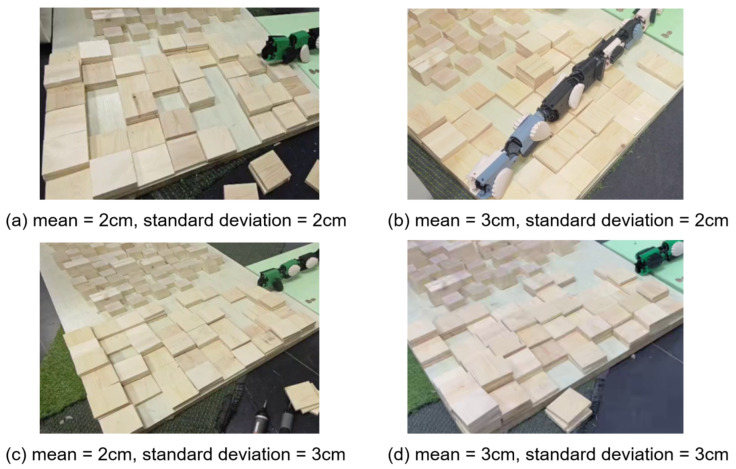
Four types of rough terrain were created based on normal distributions with varying means and standard deviations, simulating natural ground variations, designated as Terrains (**a**–**d**).

Experimental Setup: To evaluate the locomotion performance of the JiAo-II snake robot in complex three-dimensional environments, we followed the terrain construction methodology proposed by He et al. [26,27] and designed four sets of rough terrains, each measuring 72cm×48cm. These terrains consisted of ground undulations commonly found in real-world outdoor environments, as shown in Figure 18, with the terrains ordered as Terrains A, B, C, and D. Each terrain consisted of 54 square unit blocks, each measuring 8cm×8cm, with height variations between 0 cm and 6 cm in 1 cm increments. Additionally, the mean and variance of these blocks reflected the roughness of each terrain.

The 54 blocks of each terrain were positioned using a coordinate system. The top-right corner was defined as (1,1), while the bottom-left corner was defined as (8,6). The number of units at each height level was determined through sampling from the specified normal distribution using MATLAB version R2022b. These blocks were then spatially arranged on a two-dimensional grid to form a continuous and realistic terrain surface.

The block height assignment process incorporated a local correlation constraint to ensure smooth transitions between adjacent units and maintain physical feasibility. Starting from coordinate (1,1), where a base block of 0cm height was placed as the reference point, the algorithm iteratively filled the remaining positions by considering the heights of neighboring blocks based on Manhattan distance. At each step, candidate heights for the current position were generated by computing differences relative to existing neighbors, filtering out those exceeding a maximum allowable height difference threshold of 3cm, and then reconstructing valid values from the filtered set. A height was then randomly selected from this final candidate set and assigned to the corresponding block. This sequential placement proceeded along the *x*-axis and advanced row by row until the entire grid was completed.

This terrain modeling approach was specifically designed to simulate irregular surfaces with naturalistic topographic features, enabling more realistic assessment of the robot’s traversal capabilities under complex environmental conditions. By integrating statistical height distribution with spatial continuity constraints, the generated terrain provided a reproducible and reliable experimental platform for analyzing the performance of JiAo-II under various gait configurations.

**Figure 19:**
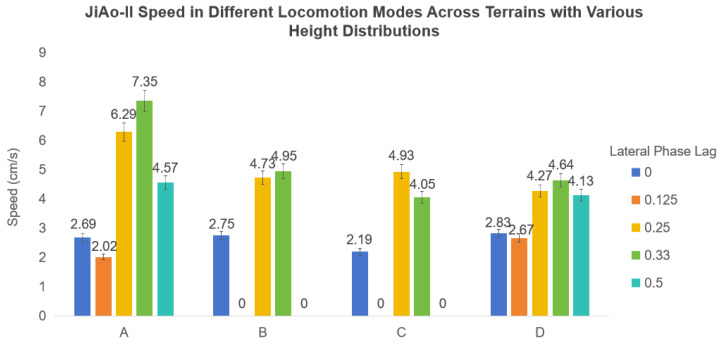
The speed of the robot employing wheel–body coordinated locomotion was measured as it traversed rough terrains under varying parameter settings. The speed of 0 cm/s indicated that the robot failed to cross the terrain.

Experimental Process and Result Analysis: Experiments were conducted for each terrain setup and wheel phase difference configuration. In each trial, both the crossing speed and success rate were recorded, and the final speed results were averaged to enhance statistical confidence. The experimental outcomes were summarized in Figure 19. In Terrains A and D, the robot successfully traversed all conditions. However, in Terrains B and C, when the lateral phase lag was set to 0.125 and 0.5, the robot failed to complete the traversal. Its stability was significantly compromised by the environment, increasing the likelihood of tipping over. In contrast, the remaining three phase lag settings demonstrated superior stability performance.

**Figure 20:**
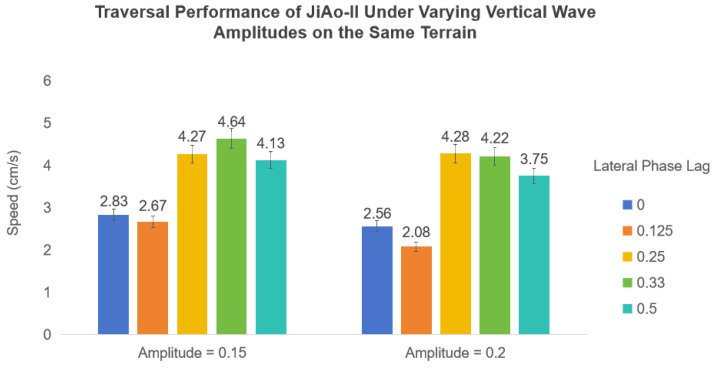
The locomotion performance of JiAo-II was tested under varying vertical wave amplitudes over the same terrain.

Additionally, we investigated the effect of vertical wave amplitude on the traversal performance of JiAo-II on the same terrain by adjusting its waveform parameters. As shown in Figure 20, we observed that when the vertical wave amplitude was too small, JiAo-II failed to traverse the terrain successfully. When the amplitude was excessively large, both stability and traversal speed deteriorated. Only with an appropriate vertical wave amplitude did JiAo-II achieve optimal performance, demonstrating superior stability and higher locomotion speed simultaneously.

## 5. Conclusions

This article introduced JiAo-II, a novel snake robot equipped with elliptical wheels that effectively combined the advantages of wheeled locomotion and body-based locomotion while addressing their inherent limitations. Three locomotion modes were implemented based on the innovative elliptical wheels. The three basic mobile modes were extensively tested indoors and outdoors, and the efficiency of the three modes was compared and analyzed. The integrated wheels significantly enhanced JiAo-II’s mobility and speed on flat surfaces. When encountering obstacles or navigating confined spaces, JiAo-II switched to body-based locomotion mode, utilizing its flexibility to overcome these challenges. In this way, JiAo-II dynamically adjusted its locomotion strategy in response to environmental changes, demonstrating exceptional terrain adaptability and flexible operational methods. Currently, mode transitions are executed through pre-programmed control strategies.

However, several challenges were encountered during the experiments. The limited load-bearing capacity of individual joints led to gear slippage and wear. Additionally, due to the increased overall weight of the robot, it was no longer able to complete the climbing task on a vertical cylindrical pipe. To address these issues, future research will focus on two key directions. First, improving the structural strength and stability of the system to resolve joint overload and gear slippage problems, while enhancing the robot’s resistance to toppling on uneven or inclined terrains. Potential solutions include optimizing joint design, increasing the reliability of the transmission system, enlarging the support area, and selecting higher-performance actuators. Second, enhancing the robot’s autonomy by developing robust environmental perception modules and real-time decision-making algorithms to enable seamless and intelligent transitions between locomotion modes, thereby reducing dependence on pre-programmed instructions. These improvements are expected to significantly enhance the robustness, adaptability, and intelligence of JiAo-II, advancing it toward a high-performance mobile platform capable of autonomous operation in diverse and unstructured environments.

## Figures and Tables

**Table 1:** Comparison of locomotion capabilities.

Related Work	Image	Tree Climbing	Helix Rolling	Lateral Rolling	Gap Crossing
Takemori et al., 2022 [6]	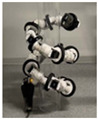	✓	✓	✓	×
Hatton et al., 2010 [8]	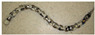	✓	✓	✓	×
Takaoka et al., 2011 [13]	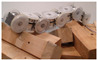	×	×	✓	✓
Kamegawa et al., 2004 [15]	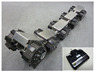	×	×	×	✓
Chong et al., 2023 [19]	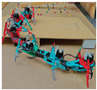	×	×	×	✓
Proposed method	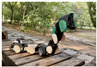	✓	✓	✓	✓

**Table 2:** Hardware platform specifications of JiAo-II.

Subject	Type	Parameter
Unit module	Diameter	7.9 cm
	Length	12.1 cm
	Mass	0.278 kg
Snake robot	Length	105.7 cm
	Mass	2.231 kg
Motor	Model	Dynamixel XH430-W350-R
	Stall Torque	4.1 Nm

**Table 3:** We measured the moving speed of JiAo-II under body-based locomotion and with different lateral phase lags for wheeled locomotion.

Locomotion Mode	Body-Based Locomotion	Wheeled Locomotion				
	Helix Rolling Gait	$\Phi_{\mathrm{lat}}=0$	$\Phi_{\mathrm{lat}}=0.125$	$\Phi_{\mathrm{lat}}=0.25$	$\Phi_{\mathrm{lat}}=0.33$	$\Phi_{\mathrm{lat}}=0.5$
Speed (cm/s)	0.90	3.43	2.55	5.43	5.83	5.11

**Table 4:** The speed of JiAo-II when crossing simple obstacles using the wheel-body coordinated locomotion.

Lateral Phase Lag	0	0.125	0.25	0.33	0.5
Speed (cm/s)	2.01	1.89	3.84	3.35	3.03

## Data Availability

The original contributions presented in this study are included in the article.
